# Ezh2 Control of Bivalent Genes Fine-Tunes Developmental Competence During Retinogenesis

**DOI:** 10.1167/iovs.67.6.18

**Published:** 2026-06-10

**Authors:** Emily Davis, Abdullah Khan, Issam Aldiri

**Affiliations:** 1 Departments of Ophthalmology, University of Pittsburgh School of Medicine, Pittsburgh, Pennsylvania, United States

**Keywords:** enhancers, polycomb, progenitor cells, chromatin, PRC2

## Abstract

**Purpose:**

Chromatin-based repression is essential for retinal development, yet the genome-wide binding landscape of Polycomb repressive complex 2 (PRC2) in the developing retina has not been defined. In particular, how the PRC2 catalytic subunit EZH2 associates with chromatin and relates to transcriptional control in retinal progenitor cells (RPCs) remains incompletely characterized.

**Methods:**

Genome-wide EZH2 binding and H3K27me3 deposition were profiled in the developing mouse retina and integrated with transcriptomic analyses following conditional *Ezh2* deletion in RPCs. Chromatin state annotations and enhancer–promoter interaction data were used to examine regulatory features associated with EZH2-bound loci.

**Results:**

EZH2 binding was enriched at promoter regions and strongly associated with H3K27me3-marked chromatin. Integration with transcriptomic data revealed that genes upregulated following Ezh2 loss were frequently EZH2-bound and enriched for loci exhibiting overlapping H3K27me3 and H3K4me3 signals at the population level. Among upregulated genes, those bound by EZH2 showed greater fold-change magnitude than unbound genes, whereas H3K27me3 occupancy alone did not differentiate fold-change within the upregulated set. Analysis of transcription factor-occupied loci indicated partial overlap between retinal progenitor transcriptional networks and Polycomb-marked regions. At the *Pitx2* locus, EZH2 and H3K27me3 occupancy coincided with a distal enhancer that is accessible and transcriptionally competent in the developing retina.

**Conclusions:**

These findings provide a genome-wide characterization of EZH2 occupancy in the developing mouse retina and support a model in which EZH2-mediated repression is associated with reduced transcription from chromatin that otherwise displays features of transcriptional competence. This work offers a framework for understanding how Polycomb-associated chromatin states intersect with retinal transcriptional programs to maintain lineage-appropriate gene expression.

Retinogenesis is a highly conserved developmental process in which a pool of multipotent retinal progenitor cells (RPCs) proliferate and differentiate in a spatiotemporal manner.^1^^,^^2^ Retinal differentiation occurs in two distinct waves, with early-born cell types (retinal ganglion cells, cones, amacrine cells, and horizontal cells) generated first during embryonic development, followed by late-born cell types (bipolar cells, rods, and Muller glia). This process is achieved through dynamic gene regulatory networks that activate and repress genes in a coordinated manner to generate the seven cell types in the appropriate space, at the appropriate time, and in the correct proportions. These gene expression dynamics are regulated by the complex interplay between retinal transcription factors (TFs) and chromatin dynamics that poise genes for activation.^3^ One way to regulate chromatin structure is through the post-translational modification (PTM) of histones, which may alter gene expression by changing DNA accessibility to TFs and transcriptional machinery. How chromatin regulators, particularly those implicated in gene repression, affect retinal development remains poorly understood.

The Polycomb group genes (PcGs) play an essential role in embryonic development by maintaining a stem cell-specific gene signature and regulating differentiation through repressive histone modifications.^4^^,^^5^ PcGs were initially identified in Drosophila and are highly conserved across evolution, specifically in their role in regulating Hox gene expression.^6^ PcGs form the histone-modifying complexes PRC1, which is responsible for monoubiquitination of H2K119, and PRC2, which catalyzes the di- and trimethylation of lysine 27 on histone H3 (H3K27me2/3) via its core subunit Enhancer of Zeste Homolog 2 (EZH2). Homolog of EZH2, EZH1, also has the capacity to mediate trimethylation and, similar to EZH2, is required for regulating differentiation of ESCs.^7^^–^^9^ EZH2 cannot function in isolation because it requires the other core subunits of PRC2, SUZ12, and EED, for enzymatic activity.^10^^,^^11^ PRC2 activity is essential for early embryonic development, as loss of core PRC2 subunits is embryonically lethal.^11^ Upon loss of PRC2 subunits, in various developmental contexts, Hox gene repression is aberrantly upregulated.^12^^–^^14^

One important mechanism by which PRC2 regulates developmental gene expression is through the establishment of bivalent chromatin domains, defined by the co-occurrence of the repressive histone mark H3K27me3 and the activating mark H3K4me3 at gene promoters.^15^ In ESCs, the presence of bivalent domains result in silenced expression of developmental genes, allowing chromatin-based maintenance of repression.^15^^–^^17^ This chromatin configuration provides a flexible regulatory state in which genes remain repressed in multipotent cells but can be swiftly activated upon removal of H3K27me3 as differentiation proceeds.^4^^,^^5^ Poised states are particularly important in tissues that require precise temporal control of cell fate decisions, allowing progenitor cells to maintain developmental potential while preventing premature or inappropriate gene expression.

PRC2 has been shown to play essential roles across multiple developing tissues and is a critical regulator of neurogenesis.^9^^,^^11^^,^^18^^,^^19^ During mouse retinogenesis, the PRC2 core component Ezh2 is highly expressed in RPCs, whereas its homolog Ezh1 is expressed at lower levels.^13^^,^^20^ PRC2 function has been investigated through conditional deletion of core subunits, including *Ezh2* and *Eed*, in RPCs.^13^^,^^21^^,^^22^ Loss of *Ezh2* in RPCs result in severe defects in retinal lamination, aberrant cell fate specification, and a profound loss of progenitor proliferation.^13^^,^^22^ Transcriptomic analyses of *Ezh2*-deficient retinas further revealed upregulation of genes that are normally silenced during retinal development, many of which are associated with non-retinal lineages.^13^ Conditional loss of *Ezh2* also results in the degeneration of photoreceptors due to the loss of H3K27me3 at the *Six1* promoter, leading to aberrant upregulation in photoreceptors and driving progressive degeneration.^23^ This ultimately links developmental chromatin modifications to the maintenance of healthy retinal cell types. Despite these insights into EZH2 function and its transcriptional consequences, there has been no data on EZH2 genomic occupancy during normal retinal development in any species. Elucidating EZH2 genomic target loci in the retina is essential for understanding its role in retinal development and disease.

Here, we characterized EZH2 genomic occupancy in the developing mouse retina. EZH2 binding was predominantly localized to promoter regions of target genes and frequently co-occurred with the repressive histone mark H3K27me3. Integration of EZH2 binding profiles with H3K27me3 enrichment and transcriptomic data following *Ezh2* loss indicated that many EZH2-targeted genes exhibit increased expression upon *Ezh2* perturbation. We also examined the relationship between EZH2-associated chromatin and RPC transcriptional networks, identifying partially distinct patterns of association. Collectively, these data support a role for EZH2-associated chromatin in modulating gene expression programs linked to progenitor cell states and in limiting expression of genes not normally active during retinogenesis.

## Methods

### Animals

C57BL/6J mouse strain (Jax lab; 000664) was used in ChIP. All mice were maintained in accordance with the guidelines set forth by the Institutional Animal Care and Use Committee (IACUC) of the University of Pittsburgh.

### Chromatin Immunoprecipitation 

Chromatin immunoprecipitation (ChIP) was prepared as previously described.^24^ In brief, retina tissue was dissected from the C57BL/6J mouse strain, incubated with 1% formaldehyde, and fixation was stopped by the addition of 0.125 M glycine. Chromatin was isolated by adding lysis buffer, and sonication was performed to shear DNA to an average fragment size of 200 to 1000 bp. Chromatin were precleared with protein G agarose beads and immunoprecipitations were performed using two different antibodies specific to Ezh2 (Active Motif # 39901 and Cell Signaling Technologies # 5246). H3K27me3 ChIP was performed using Active Motif # 39156 antibody. After washing, immune complexes were eluted from the beads using SDS buffer, treated with RNase and proteinase K, and de-crosslinked by overnight incubation at 65°C. ChIP DNA was then purified and DNA sequencing libraries were prepared using NEB DNA Library Prep Kit, following the manufacturers’ protocols. Libraries were sequenced on the Illumina platform (Illumina NextSeq 2000).

### ChIP-Sequencing Processing and Peak Calling

Paired-end ChIP-sequencing (ChIP-seq) reads were processed using the nf-core/chipseq pipeline (version 2.0.0)^25^ executed with Nextflow (version 23.10.0).^26^ Read quality control was performed with FastQC (version 0.11.9), and adapter/quality trimming was carried out with Trim Galore (version 0.6.7)/cutadapt (version 3.4). Reads were aligned to the mouse reference genome (mm10) using Bowtie2 (version 2.4.4).^27^ Post-alignment processing was performed using SAMtools (version 1.1.1)^28^ for BAM handling and Picard (version 2.27.4) for duplicate marking. Marked duplicate reads were subsequently removed using bamtools (version 2.5.2)^29^ with default filtering parameters. Peaks were called using MACS2 (version 2.2.7.1)^30^ in broadPeak mode with a broad cutoff of *P* < 1 × 10^-4. Comparison of AM and CST antibody datasets demonstrated moderate agreement (Pearson correlation = 0.50–0.56), with 1123 peaks identified as peaks present in both datasets (Supplementary Fig. S1A). However, one of the AM samples exhibited a very high duplication rate (>60%), thus peaks from the CST antibody were exclusively selected for downstream analysis.

### ATAC-Seq Processing, Alignment, and Peak Calling (Wild-Type Time Course)

Wild-type (WT) developmental time-course ATAC-seq data were obtained from the NCBI Gene Expression Omnibus (GEO) SuperSeries GSE87064.^31^ Paired-end WT ATAC-seq reads were processed using the nf-core/atacseq pipeline (version 2.0)^25^ executed with Nextflow (version 23.10.1). Read quality control was performed with FastQC (version 0.11.9),^32^ and adapter/quality trimming was performed using Trim Galore (version 0.6.7) / cutadapt (version 3.4). Reads were aligned to the mouse reference genome (mm10) using Bowtie2 (version 2.4.4). Post-alignment processing (filtering, sorting, and indexing) was performed using SAMtools (version 1.16.1) and bamtools (version 2.5.2), with duplicate marking and BAM merging performed using Picard (version 2.27.4). Reads overlapping blacklist regions and duplicates were removed using BEDTools (version 2.30.0) as implemented in the pipeline default filtration settings.

Accessible chromatin peaks were called using MACS2 (version 2.2.7.1) in narrowPeak mode with a significance threshold of *P* < 1 × 10^-5. Peak annotation was performed with HOMER (version 4.11).^33^ Library-level quality control (QC) metrics were generated within the pipeline, including transcription start site (TSS) enrichment and related ATAC-seq QC summaries using ataqv (version 1.3.0) and FRiP calculations using BEDTools (version 2.30.0).

### RNA-Seq Processing, Quantification, and Differential Expression Analysis

Publicly available RNA-seq datasets were obtained from the NCBI GEO. The *Ezh2* conditional knockout (cKO) retina RNA-seq dataset was downloaded from GSE65082 (E16.5; 4 controls and 4 cKO samples).^13^ The WT developmental time-course dataset was obtained from GSE87064. Paired-end RNA-seq reads were processed using the nf-core/rnaseq pipeline (version 3.14.0) executed with Nextflow (version 25.04.6). Read quality control was performed with FastQC (version 0.12.1), and adapter/quality trimming was carried out using Trim Galore (version 0.6.7) / cutadapt (version 3.4). Reads were aligned to the mouse reference genome (mm10) using STAR (version 2.7.9a), with BAM processing and indexing performed using SAMtools (version 1.17). Duplicate reads were marked using Picard (version 3.0.0) and subsequently removed.

Transcript abundance quantification was performed using Salmon (version 1.10.1). Transcript-level estimates were summarized to gene-level counts using tximeta/tximport (tximeta version 1.12.0), producing a gene-level count matrix for downstream analyses.

Gene-level count matrices generated from Salmon quantifications (via tximeta/tximport) were used for downstream differential expression analysis in DESeq2. The DESeq2 size-factor normalization was applied to account for library-size differences, and dispersion parameters were estimated using the standard DESeq2 framework. Differential expression was assessed using DESeq2 model fits with design formulas matched to the experimental comparisons (e.g., approximate condition for *Ezh2* cKO versus control, and timepoint/stage terms for the WT developmental series), followed by hypothesis testing using Wald tests (pairwise contrasts) or likelihood ratio tests where appropriate. The *P* values were corrected for multiple testing using the Benjamini–Hochberg procedure, and genes were considered differentially expressed based on the chosen adjusted *P* value and effect-size thresholds. For visualization and QC, normalized expression values were transformed (e.g., variance-stabilizing or regularized log transformation) and used for sample-level exploratory analyses such as heatmaps.

### Signal Track Generation and Downstream Visualization

Genome-wide signal tracks were generated from processed BAM files using deepTools bamCoverage (version 3.5.6) to create bigWig files normalized to RPGC (reads per genomic content). The bigWig signal tracks were then used with multiple peak sets for downstream plotting and visualization, including signal aggregation and heatmap/profile-style summaries using deepTools (computeMatrix/plotHeatmap/plotProfile). Functional annotation of promoter peaks was done using ChIPseeker, defining peaks to genes within 2kb of the TSS.

### Statistical Analysis

Statistical analysis information for each figure is detailed in the figure legends with the number of samples (*n*), the *P* value used, and the data presentation described. For the quantification of signal intensity plots, two-sample *t*-test (unpaired, *P* < 0.05) was used, with boxplots showing the counts per million (CPM)-normalized values. Boxplots are drawn using the median to define interquartile ranges (IQR), with outliers defined as ±1.5 IQR from the third quartile or first quartile, respectively. For comparison of log2 fold changes, statistical tests and data visualization was performed on Prism (version 10.4.1) using the Wilcoxon test (*P* < 0.05). Data visualized in Tukey Boxplots, where the median is a horizontal line, the box is the IQR, and outliers are defined as ±1.5 IQR from the third quartile or first quartile.

## Results

### EZH2 Genomic Occupancy Profiled During Mouse Retinogenesis

**Figure 1.**
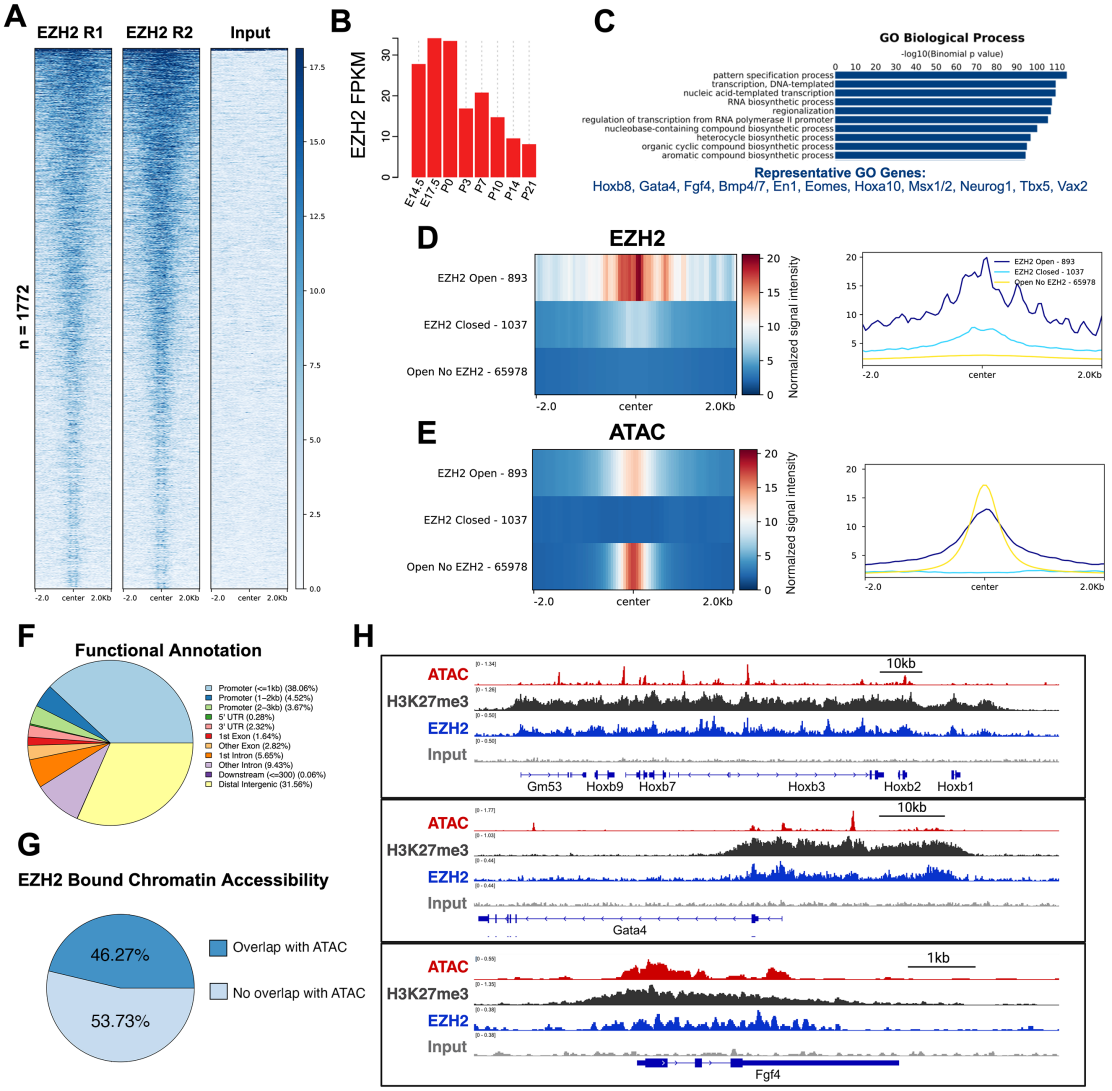
**EZH2 genomic occupancy profiled and characterized in E14.5 developing retina.** (**A**) EZH2 ChIP-seq replicates and input binding signal intensity plotted ± 2kb from the center of the peak. (**B**) Bar graph showing Ezh2 RNA level (FPKM) across developmental stages. (**C**) GO biological processes analysis of EZH2 peaks with selected representative genes shown. (**D,** **E**) Representative compressed heatmap (*left*) and average plot (*right*) showing signal intensity for EZH2 ChIP **D** and ATAC-seq **E**. (**F**) Pie chart of genomic distribution of EZH2 peaks. (**G**) Pie chart showing the proportion of EZH2 peaks that overlap with ATAC peaks. (**H**) Genome browser track highlighting EZH2 binding, H3K27me3 enrichment, and ATAC at E14.5. Input shown as control for EZH2.

Ezh2 is expressed in RPCs and declines postnatally as neurogenesis proceeds (Fig. 1B). To identify genome-wide PRC2 occupancy during retinogenesis, we performed ChIP-seq for EZH2 in mouse embryonic (E) 14.5 retina using two different EZH2 antibodies (*n* = 2 per condition; Methods). Reads were filtered and aligned to the mouse reference genome (mm10), then peaks were called using MACS2 for broad peaks. Visual inspection indicated that both antibodies produced concordant binding profiles (see Supplementary Fig. S1); however, based on QC metrics (see Methods; Supplementary Table S1), the CST antibody was used for all subsequent analyses. EZH2 ChIP identified 1772 binding sites common in both CST antibody replicates, with functional annotation indicating that EZH2 primarily binds promoter regions (38.06%) and distal intergenic regions (31.56%; Figs. 1A, 1F; Supplementary Table S2). Gene Ontology (GO) analysis of peaks using the Genomic Regions Enrichment of Annotations Tool (GREAT)^34^ revealed enrichment for terms such as pattern specification, transcription, and regionalization, suggesting that EZH2 preferentially targets regulatory regions associated with developmental patterning and transcriptional control in the developing retina (Fig. 1C; see Supplementary Table S2). Representative targets include developmental regulators such as Hox genes (e.g., *Hoxb8*), lineage-defining TFs (e.g., *Gata4*), and key signaling molecules (e.g., *Fgf4*), all of which are not expressed in the developing mouse retina (see Figs. 1C, 1H).

EZH2 is associated with chromatin compaction and gene repression.^9^ To investigate how EZH2 binding relates to chromatin accessibility in the developing retina, we overlaid EZH2 occupancy with ATAC-seq data from WT mouse E14.5 retina using Bedtools. We found that 46.27% of EZH2-bound regions overlap with ATAC-seq peaks (Fig. 1G; Supplementary Table S3). Notably, EZH2 exhibits stronger binding at ATAC-overlapping regions compared with ATAC-non-overlapping regions (Fig. 1D). Consistent with this, EZH2-bound ATAC-overlapping regions display reduced ATAC signal relative to regions lacking EZH2 binding (Fig. 1E), indicating that EZH2 occupancy is associated with diminished accessibility within otherwise accessible chromatin.

### EZH2 Targets Poised and Repressed Genes via H3K27me3 in the Developing Retina

**Figure 2.**
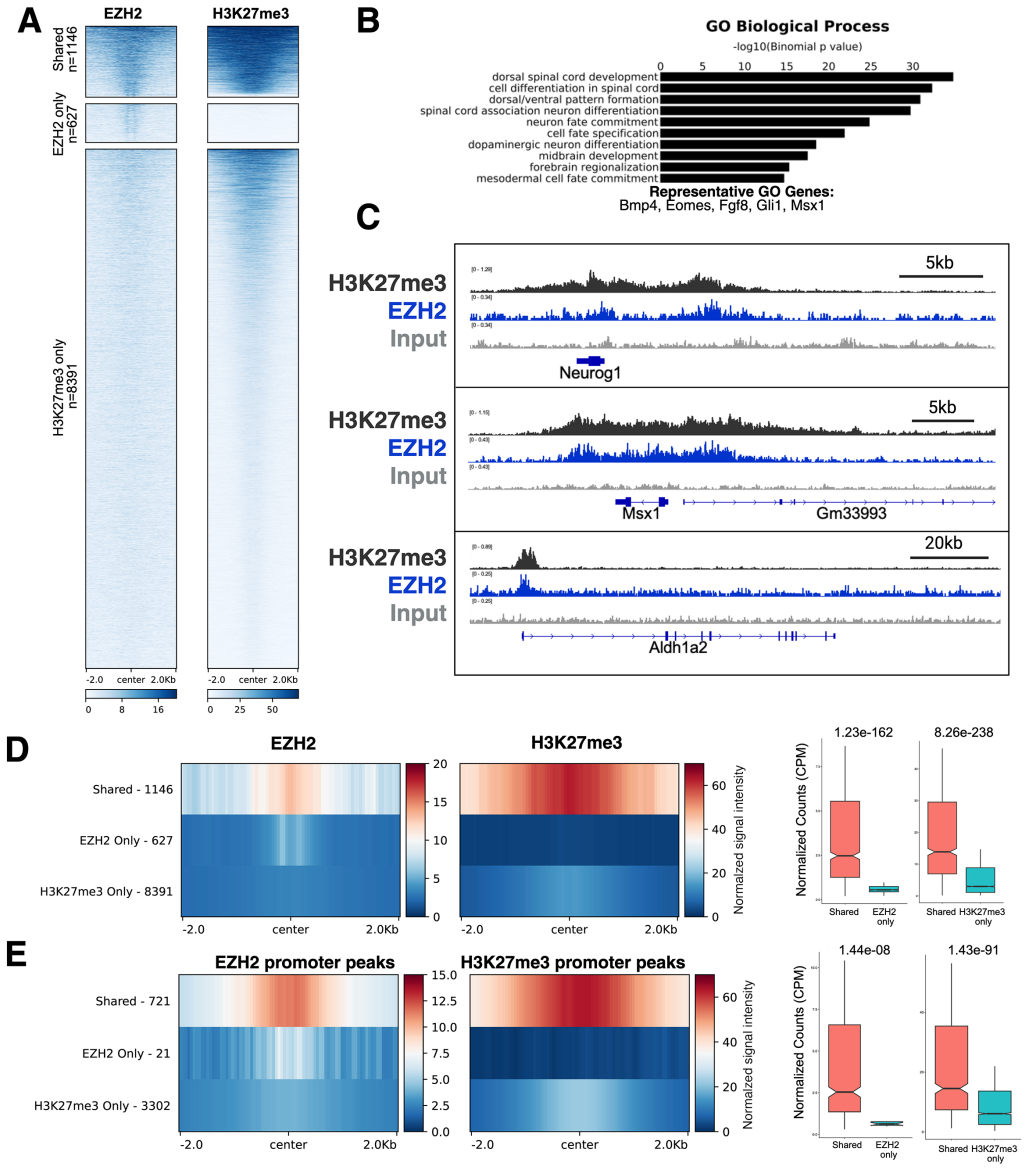
**EZH2 and H3K27me3 have shared genomic occupancy during retinogenesis.** (**A**) Heat plots showing H3K27me3 and EZH2 shared and unique peaks (plotted ± 2kb from the center of the peak). (**B**) GO biological processes analysis of H3K27me3 peaks with selected representative genes shown. (**C**) Genome browser track highlighting EZH2 and H3K27me3 co-enrichment. Input shown as control for EZH2. (**D**) Representative compressed heatmap (*left*), average plot (*middle*), and normalized read counts (*right*) showing signal intensity for EZH2 and H3K27me3, showing shared peaks having stronger binding intensity. (**E**) Representative compressed heatmap (*left*), average plot (*middle*), and normalized read counts (*right*) showing signal intensity for EZH2 and H3K27me3 promoter only peaks, showing shared promoter peaks having stronger binding intensity.

Because EZH2 is responsible for depositing the histone repressive mark H3K27me3,^35^ we assayed H3K27me3 occupancy in the developing retina using ChIP-seq performed at E14.5 (*N* = 2; Methods). As previously shown, H3K27me3 is highly enriched in Hox gene clusters, demonstrating successful ChIP (see Fig. 1H; Supplementary Fig. S1B). GO analysis revealed enrichment for terms associated with neuron fate commitment and cell fate commitment (Figs. 2B, 2C). We then compared the genomic profiles of H3K27me3 and EZH2. We found a strong correlation (>80%) between both, with a substantial fraction of EZH2 peaks (1146/1773, 64.6%) coinciding with H3K27me3, consistent with EZH2’s canonical role in depositing this repressive modification (Fig. 2A; see Supplementary Fig. S1A). Interestingly, signal intensity analysis revealed that EZH2 binding is stronger at shared regions than at EZH2-only regions (Fig. 2D; Wilcoxon test, *P* value = 1.23e–162). Likewise, we found that H3K27me3 chromatin binding is more enriched at shared sites than at loci without EZH2 (see Fig. 2D; Wilcoxon test, *P* value = 8.26e–238). Because a substantial proportion of EZH2 occupancy occurs within promoters, we extended this analysis to promoter-only regions (Supplementary Table S4). Almost all EZH2 promoter-only peaks were co-occupied with H3K27me3 (721/742, 97.1%), and the increased chromatin-binding intensity at shared sites was also observed at the promoter level (Fig. 2E; Wilcoxon test, *P* value < 0.05).

### Putative Bivalent Genes Marked by EZH2 Show a Tendency Toward Increased Derepression

**Figure 3.**
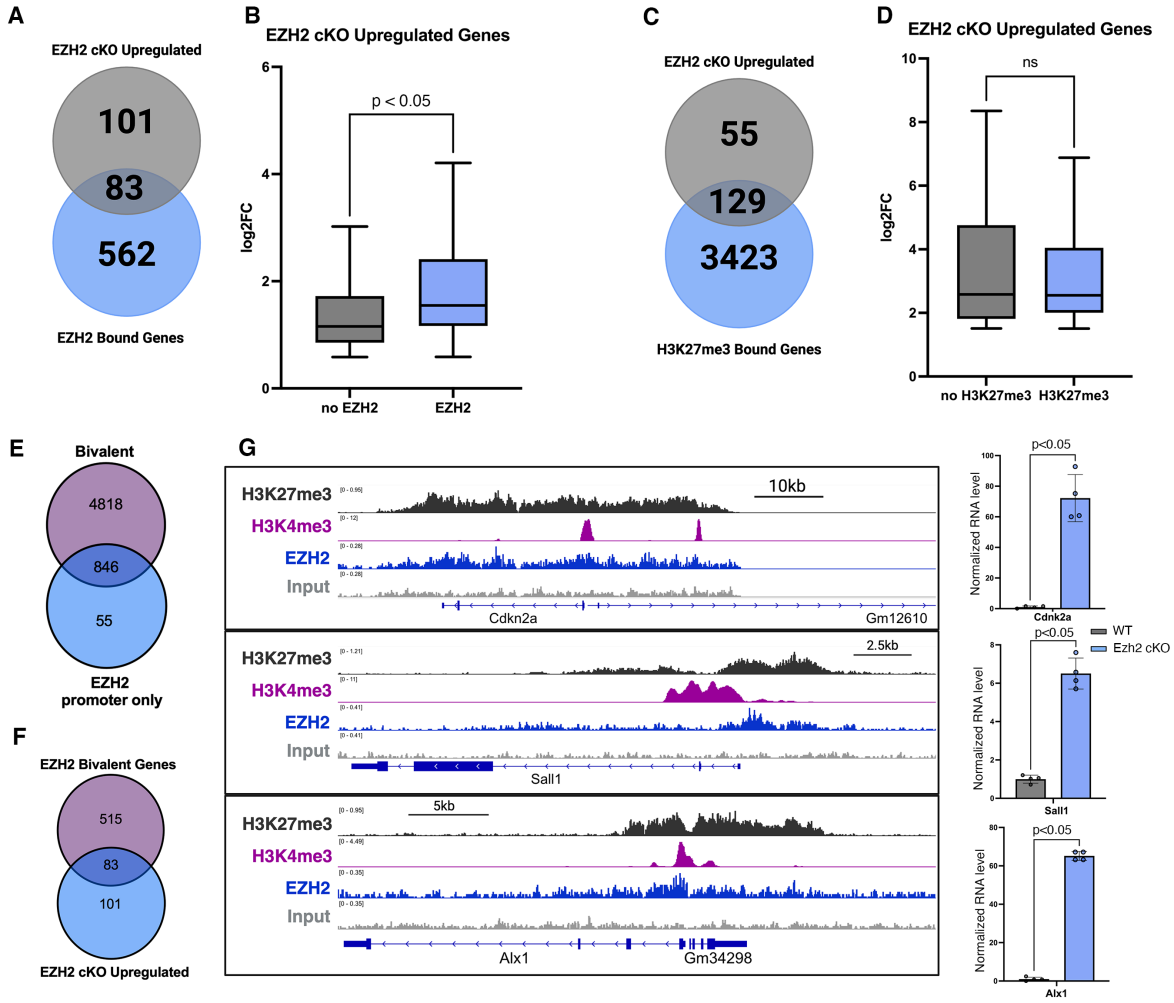
**Derepression of EZH2 targets at EZH2-bound and putative bivalent promoters.** (**A**) Intersection of EZH2 promoter-bound genes with EZH2 cKO upregulated genes. (**B**) Boxplot comparing log2 fold-changes of EZH2-bound versus unbound EZH2 cKO upregulated genes. Wilcoxon test (*P* value < 0.05). (**C**) Intersection of H3K27me3 promoter-bound genes with *Ezh2* cKO upregulated genes. (**D**) Boxplot comparing log2 fold-changes of H3K27me3-bound versus unbound EZH2 cKO upregulated genes. Wilcoxon test (ns). (**E**) Intersection of bivalent regions EZH2 promoter peaks. (**F**) Intersection of EZH2 bivalent genes and *Ezh2* cKO upregulated genes. (**G**) Genome browser track highlighting EZH2-bound promoters with putative bivalent chromatin signatures of genes that are significantly upregulated in Ezh2 cKO. Corresponding normalized counts from WT and *Ezh2* cKO on the *right*.

To explore how EZH2 coordinates transcriptional repression during retinal development, we performed an integrative analysis of its genomic occupancy, H3K27me3 deposition, and downstream transcriptional targets, leveraging publicly available bulk RNA-seq data from *Ezh2* cKO at E16.5 of the developing retina.^13^ *Ezh2* cKO resulted in the upregulation of 184 and the downregulation of 14 genes (fold change [FC] = >1.5, adjusted *P* value < 0.05; Methods). We found that EZH2 binds the promoters of 45.10% (83/184) of the genes that are significantly upregulated (Fig. 3A; Supplementary Table S5). Importantly, in the *Ezh2* cKO retina, EZH2-bound genes exhibit a greater degree of upregulation than unbound genes (Fig. 3B; Wilcoxon test, *P* < 0.05). In comparison, H3K27me3 occupies the promoters of 70.10% (129/184) of upregulated genes (Fig. 3C). However, H3K27me3-bound upregulated genes were not more significantly upregulated than non-H3K27me3-bound genes (Fig. 3D; Wilcoxon test, *P* value = 0.9687).

Bivalently marked chromatin primes genes for subsequent activation upon removal of H3K27me3.^15^^,^^36^ To investigate this in the developing retina, we examined the bivalent state of EZH2-bound regions, using previously published data that include ChIP-seq profile of H3K4me3 at E14.5 retina.^31^ We found that 93.89% of EZH2-occupied promoters (846/901 peaks), corresponding to 593 genes, are marked with putative bivalent domains (Fig. 3E; Supplementary Table S6). Interestingly, nearly half (45.10%, 83/184) of *Ezh2* cKO upregulated genes were associated with putative bivalent signatures and EZH2-bound, highlighting EZH2’s role in maintaining repression at poised genes (Fig. 3F; see Supplementary Table S6). Some examples include *Cdkn2a*, *Sall1*, and *Alx1*, which are all significantly upregulated and display putative bivalent chromatin and EZH2 occupancy on their promoter in the developing retina (Fig. 3G).

### Association Between RPC Transcription Factors and EZH2 in the Developing Retina

**Figure 4.**
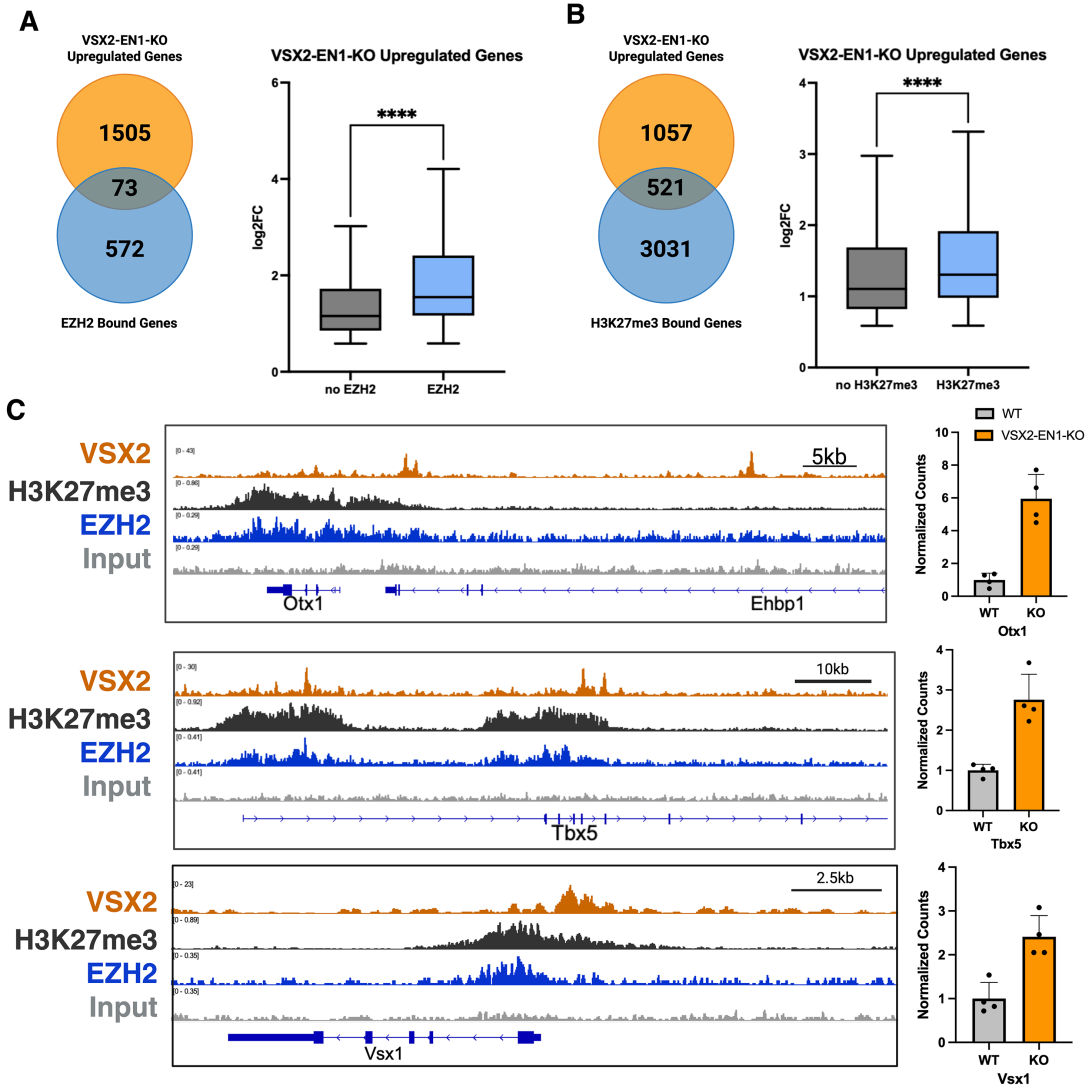
**VSX2 repression is sensitive to Polycomb-mediated repression at target gene promoters.** (**A**) Intersection of EZH2 promoter-bound genes with Vsx2-EN1-KO upregulated genes (*left*) and boxplot comparing log2 fold-changes of EZH2-bound versus unbound Vsx2-EN1-KO upregulated genes (*right*). Wilcoxon test (*P* < 0.05). (**B**) Intersection of H3K27me3 promoter-bound genes with Vsx2-EN1-KO upregulated genes (*left*) and boxplot comparing log2 fold-changes of H3K27me3-bound versus unbound Vsx2-EN1-KO upregulated genes (*right*). Wilcoxon test (*P* < 0.05). (**C**) Genome browser track highlighting VSX2, EZH2, and H3K27me3 occupancy of genes that are significantly upregulated in Vsx2-EN1-KO. Corresponding normalized counts from WT and Vsx2-EN1-KO on the *right*.

TFs can use multiple mechanisms of repression, such as binding DNA to physically block the transcriptional machinery or recruiting co-repressors.^37^ One possible mechanism of repression is the recruitment of chromatin-remodeling complexes to alter the chromatin conformation to a repressive state.^33^^–^^35^ In the developing retina, multiple RPC TFs can function as activators or repressors, depending on context, with chromatin-mediated mechanisms that remain poorly investigated. Among those is VSX2, a homeodomain-containing TF expressed in RPCs and essential for their proliferation and proper differentiation. Loss of *Vsx2* function results in a microphthalmic eye.^38^^–^^40^ We investigated whether VSX2-mediated repression may be partially mediated by PRC2 recruitment and H3K27me3 deposition at selective VSX2-occupied loci targeted for repression. First, we determined the extent by which VSX2 occupies H3K27me3-labeled loci, leveraging ChIP-seq data performed on WT mouse retina at E14.5.^24^ We found that 579 peaks are shared (Supplementary Table S7). We then used the Vsx2-EN1-KO mouse model, in which an enhancer required for embryonic *Vsx2* expression has been deleted, resulting in a significant reduction in Vsx2 and loss of RPC proliferation potential.^24^ EZH2 binding was detected at only a small fraction of Vsx2-EN1-KO significantly upregulated genes 4.62% (73/1578), whereas H3K27me3 marked a much larger proportion (33.02%, 521/1578; Figs. 4A, 4B; Supplementary Table S8). Notably, genes bound by EZH2 or enriched for H3K27me3 showed significantly greater upregulation compared to genes lacking such binding (see Figs. 4A, 4B; Wilcoxon test, *P* < 0.05). These targets include key TFs (e.g., *Tbx5*), signaling pathway components (e.g., *Bmp4/7*), and cell-cycle regulators (e.g., *Ccnd2*; Fig. 4C).

Interestingly, multiple ciliary marginal zone-restricted genes that are upregulated upon loss of Vsx2 expression in the developing retina, including *Otx1*, *Bmp7*, *Ccnd2*, and *Wnt9a*, displayed VSX2 and EZH2 binding and H3K27me3 enrichment in the WT retina (see Fig. 4C).^41^^,^^42^ To further assess the extent of shared repression, we directly compared genes upregulated upon *Vsx2* loss with those upregulated following Ezh2 perturbation. This analysis revealed only 23 common targets, including Sall1, Aldh1a2, and Ccnd2, indicating limited overlap between VSX2- and EZH2-mediated repression programs (Supplementary Figs. S2A, S2B).

We also performed a parallel analysis on SOX2, a SOXB1-HMG box TF expressed in RPCs and essential for their neural competence.^42^^–^^44^ SOX2 and VSX2 are known to participate in a shared transcriptional network during retinal development, promoting the expression of regulators of retinal neurogenesis and repressing genes associated with non-neural cell fate.^41^^,^^45^^,^^46^ Notably, using our RNA-seq data generated from *Sox2* cKO retina,^45^ we found that several non-neural genes repressed by SOX2 exhibit H3K27me3 enrichment at their promoters. Among genes significantly upregulated following *Sox2* loss, only 1.74% (38/2180) showed EZH2 promoter occupancy (see Supplementary Table S8). However, these EZH2-bound genes showed a greater magnitude of upregulation than genes lacking EZH2 binding (Supplementary Fig. S2C; Wilcoxon test, *P* value < 0.05). Likewise, 20.0% (438/2180) of Sox2-upregulated genes were marked by H3K27me3 and were significantly more strongly upregulated compared to genes without this modification (Supplementary Fig. S2D; Wilcoxon test, *P* value < 0.05). Collectively, these findings suggest that SOX2 and VSX2 do not function as global regulators of PRC2 activity but instead may converge on PRC2-mediated repression at a restricted subset of target genes during retinal development.

### Evidence for a Putatively Primed but Repressed Pitx2 Regulatory Circuitry in the Developing Retina

We sought to further elucidate the functional mechanisms underlying the significant upregulation of genes upon loss of Polycomb-mediated repression. Loss of Ezh2 function typically results in selective derepression of only a subset of H3K27me3-targeted genes, suggesting that loss of *Ezh2* and its repressive mark is insufficient to drive robust transcriptional activation. Rather, effective gene activation also requires a permissive chromatin context in which regulatory elements are engaged by appropriate tissue-specific TFs. We therefore hypothesized that derepressed genes in *Ezh2* cKO retina are transcriptionally primed for activation via recruitment of RPC TFs, but H3K27me3 occupancy prevents such activation*.*

**Figure 5.**
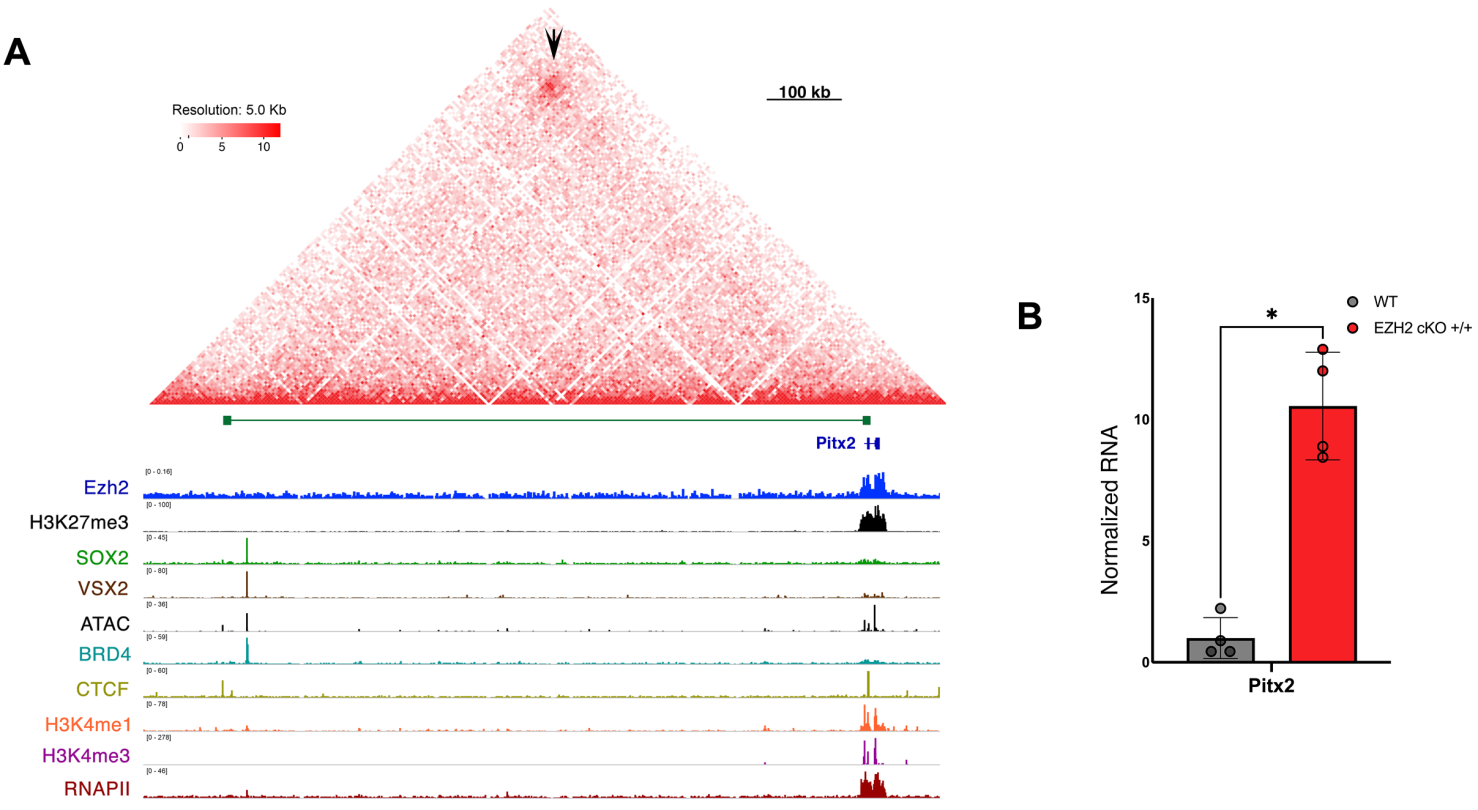
***Pitx2*** **regulatory landscape poised for activation in the absence of Polycomb-mediated repression.** (**A**) Hi-C–based genomic view of the *Pitx2* locus showing chromatin interactions and associated chromatin features. The promoter is bound by EZH2 and displays putative bivalent H3K27me3 and H3K4me3 marks. A distal enhancer located 829 kb from the transcription start site (TSS) exhibits active enhancer signatures, including ATAC-seq accessibility, BRD4, H3K4me1, and RNA polymerase II (RNAPII), and is occupied by the retinal progenitor transcription factors VSX2 and SOX2. Note the presence of CTCF, facilitating enhancer-promoter interactions. (**B**) *Pitx2* RNA level in wild-type (WT) and Ezh2 conditional knockout (cKO) developing retinas, extracted from Zhang and co-workers. *P* < 0.05.

To test this hypothesis, we examined the regulatory landscape of genes that are significantly upregulated in *Ezh2* cKO and EZH2-bound and identified Pitx2 as a strong candidate gene. Pitx2 plays a crucial role in the development of anterior eye structures (cornea and iris), and it is normally not expressed in the developing retina.^47^^–^^49^ Loss of *Ezh2* leads to a significant increase in Pitx2 expression^13^ (Fig. 5B).

To examine the chromatin signature of the *Pitx2* locus, we analyzed publicly available bulk ChIP-seq profiles from E14.5 mouse retina of H3K4me1, CTCF, BRD4, and RNAPII^31^ along with Ezh2 and H3K27me3 (Fig. 5A). Analysis revealed that its repression in the developing retina is associated with a putative bivalent state, as the promoter maintains features of active chromatin (H3K4me1, BRD4, RNAPII, and accessible chromatin) in addition to H3K27me3 (see Fig. 5A). To identify the cis-regulatory elements associated with *Pitx2*, we queried our previously generated Hi-C data^50^ from the mouse developing retina and identified a putative enhancer element located 829 kb from the *Pitx2* TSS. This region displays hallmark enhancer features (ATAC, BRD4, and H3K4me1) and strong physical interaction with the Pitx2 promoter region (see Fig. 5A). We scanned the regulatory landscape in search for binding RPC TFs and, surprisingly, found that this enhancer is bound by the RPC factors SOX2 and VSX2 (see Fig. 5A). Collectively, these data suggest a model in which polycomb-mediated repression at the Pitx2 promoter limits transcriptional activation by putatively primed distal regulatory elements. However, direct mechanistic evidence for this regulatory circuit awaits further functional perturbation studies.

## Discussion

Retinogenesis relies on tightly coordinated gene-expression dynamics, which are governed by chromatin-level regulation. Specifically, during retinal development, approximately 1000 genes show H3K27me3 changes that correlate with changes in gene expression.^31^ The repressive complex PRC2 has been well studied in developmental contexts involving ESCs and other developmental systems, yet its genome-wide binding landscape and chromatin associations in the developing retina have remained undefined. Previous work has dissected the function of catalytic subunit EZH2 during retinal development, identifying it as necessary for postnatal progenitor proliferation, proper cellular differentiation, and repression of non-retinal genes.^13^^,^^21^ In this study, we provide a comprehensive characterization of EZH2 genomic occupancy in the embryonic mouse retina and integrate these data with chromatin accessibility profiles and transcriptomic analyses following conditional Ezh2 deletion. Together, our findings offer insight into how PRC2-associated chromatin states intersect with retinal transcriptional programs during retinogenesis.

Genome-wide analysis revealed a strong correlation between EZH2 occupancy and H3K27me3 enrichment, consistent with Polycomb-mediated repression in other tissues. Importantly, genes bound by EZH2 showed greater transcriptional upregulation following *Ezh2* deletion than unbound genes, indicating that EZH2 occupancy marks loci that are sensitive to *Ezh2* loss. In contrast, although H3K27me3 occupies the promoters of most upregulated genes, the degree of upregulation did not differ significantly between H3K27me3-bound and unbound genes. These findings suggest that EZH2 occupancy may better identify loci that are transcriptionally sensitive to PRC2 perturbation in this context. Our data do not imply that EZH2 functions independently of H3K27me3, but rather that direct occupancy by PRC2 may reflect a chromatin context more permissive to transcriptional change upon loss of repression. This interpretation is consistent with prior studies showing that H3K27me3-rich regions exhibit higher EZH2 occupancy and stronger Polycomb activity than typical H3K27me3 peaks.^51^ Together, these observations highlight the value of integrating Polycomb protein occupancy with histone modification profiles.

A substantial fraction of EZH2-bound promoters were associated with putative bivalent chromatin signatures marked by both H3K27me3 and H3K4me3. Such configurations have been proposed to maintain genes in a transcriptionally restrained yet responsive state. In our dataset, nearly half of the genes upregulated following Ezh2 deletion were EZH2-bound and associated with these composite signatures, whereas genes marked solely by H3K27me3 were more often transcriptionally inert. These observations are consistent with EZH2 occupancy being enriched at loci with regulatory potential rather than at stably silenced genes. However, given that these analyses are derived from bulk tissue, the inferred bivalency should be interpreted cautiously. The apparent co-enrichment of activating and repressive marks may reflect population-level averaging across heterogeneous cell states rather than true co-occupancy within individual cells. Accordingly, we refer to these regions as putative bivalent domains, acknowledging that they represent composite chromatin states at the tissue level.

How PRC2 is recruited to specific genomic loci in the retina remains unresolved. Because PRC2 lacks intrinsic ability to bind DNA, recruitment is presumed to involve interactions with other chromatin-associated factors or transcriptional regulators. We examined the relationship between PRC2-associated chromatin and the RPC TFs VSX2 and SOX2, both of which play central roles in RPC identity and competence. Although we observed overlap between TF-regulated gene sets and Polycomb-marked loci, EZH2 occupancy was detected at only a minority of TF-responsive genes. These findings suggest that VSX2 and SOX2 are unlikely to function as global recruiters of PRC2 in RPCs. Instead, interactions between retinal transcriptional networks and Polycomb-mediated repression may be indirect, context-dependent, or restricted to a subset of shared target genes.

An important limitation of our analyses is that chromatin and transcriptional profiling were performed on bulk retinal tissue, which comprises heterogeneous populations of progenitor cells and differentiated neurons. Consequently, the observed co-enrichment of EZH2, histone modifications, and TF binding should not be interpreted as definitive evidence of simultaneous occupancy within the same genomic loci in individual cells. In particular, the apparent presence of bivalent chromatin states may be overestimated in bulk measurements, as activating and repressive marks could derive from distinct cell populations or differentiation states rather than coexisting on the same allele within a single cell. Thus, what appears as bivalency may, in part, reflect population-level averaging of mutually exclusive chromatin states. Still, the co-enrichment patterns are not random but are consistently detected at specific loci with known developmental regulation, and they align with transcriptional and chromatin accessibility profiles indicative of poised regulatory states. Moreover, the reproducibility of these signatures across datasets and their concordance with prior studies of Polycomb-mediated regulation argue that they capture meaningful regulatory configurations at the tissue level. Therefore, although bulk measurements may inflate the extent of true bivalency, they nonetheless provide a valid and informative readout of loci that are under dynamic and potentially competing regulatory influences during retinal development.

A further consideration is the temporal offset between ChIP- and RNA-seq datasets, with EZH2 ChIP-seq performed at E14.5 and RNA-seq of Ezh2 cKO derived from E16.5 retina. E16.5 tissue exhibits increased neuronal differentiation relative to E14.5, and transcriptional changes observed following Ezh2 deletion likely reflect a combination of direct regulatory effects and secondary consequences of developmental progression. This limits strict one-to-one correspondence between EZH2 occupancy and gene expression changes. Nonetheless, the concordance between EZH2-bound loci identified at E14.5 and genes upregulated at E16.5 supports a functional relationship between early Polycomb occupancy and later transcriptional outcomes, providing a coherent framework linking chromatin state to gene expression dynamics during retinogenesis.

Our integrative analyses provide insight into potential mechanisms underlying selective gene derepression following PRC2 disruption. At the *Pitx2* locus, we identified a putative distal regulatory element that physically interacts with the promoter, exhibits features of active enhancers, and is bound by RPC TFs, despite repression of *Pitx2* expression in the developing retina. These findings are consistent with a model in which Polycomb-associated chromatin coincides with a regulatory architecture that retains features of transcriptional competence. In this context, PRC2-mediated repression may act to limit transcriptional output from pre-existing enhancer–promoter interactions rather than fully preventing enhancer accessibility. Although this example is based on a single locus and does not establish a general mechanism, it highlights a potential mode of regulation at loci with permissive features. Importantly, even at this locus, direct functional validation will be required to confirm the proposed regulatory relationships.

PRC2 exists in at least two distinct configurations that share EZH2 as a core catalytic subunit.^52^^,^^53^ As such, the present data do not distinguish between contributions of distinct PRC2 complexes during retinal development. Notably, the PRC2.2-associated factor JARID2 has been reported to regulate the temporal progression of retinal progenitors through repression of Foxp1.^54^ These observations underscore the need of systematically profiling both core and auxiliary PRC2 subunits to delineate potential functional specialization of PRC2 complexes in the developing retina.

## Conclusions

This study provides the first genome-wide map of EZH2 occupancy in the developing mouse retina and integrates chromatin and transcriptional datasets to define features associated with PRC2-sensitive genes. Our findings support a model in which EZH2-mediated repression is associated with limiting expression of transcriptionally competent developmental genes during retinogenesis. These data establish a foundation for future mechanistic studies of Polycomb function in retinal development and disease.

## Supplementary Material

Supplement 1

Supplement 2

Supplement 3

Supplement 4

Supplement 5

Supplement 6

Supplement 7

Supplement 8

Supplement 9
