# Supplementary material for: Ezh2 Control of Bivalent Genes Fine-Tunes Developmental Competence During Retinogenesis
Source: Invest Ophthalmol Vis Sci. 2026 Jun 10;67(6):18. doi: 10.1167/iovs.67.6.18 (PMC13263970; doi:10.1167/iovs.67.6.18)
Supplement: Supplement 1 [file iovs-67-6-18_s001.docx]

**Supplementary Figures**

**
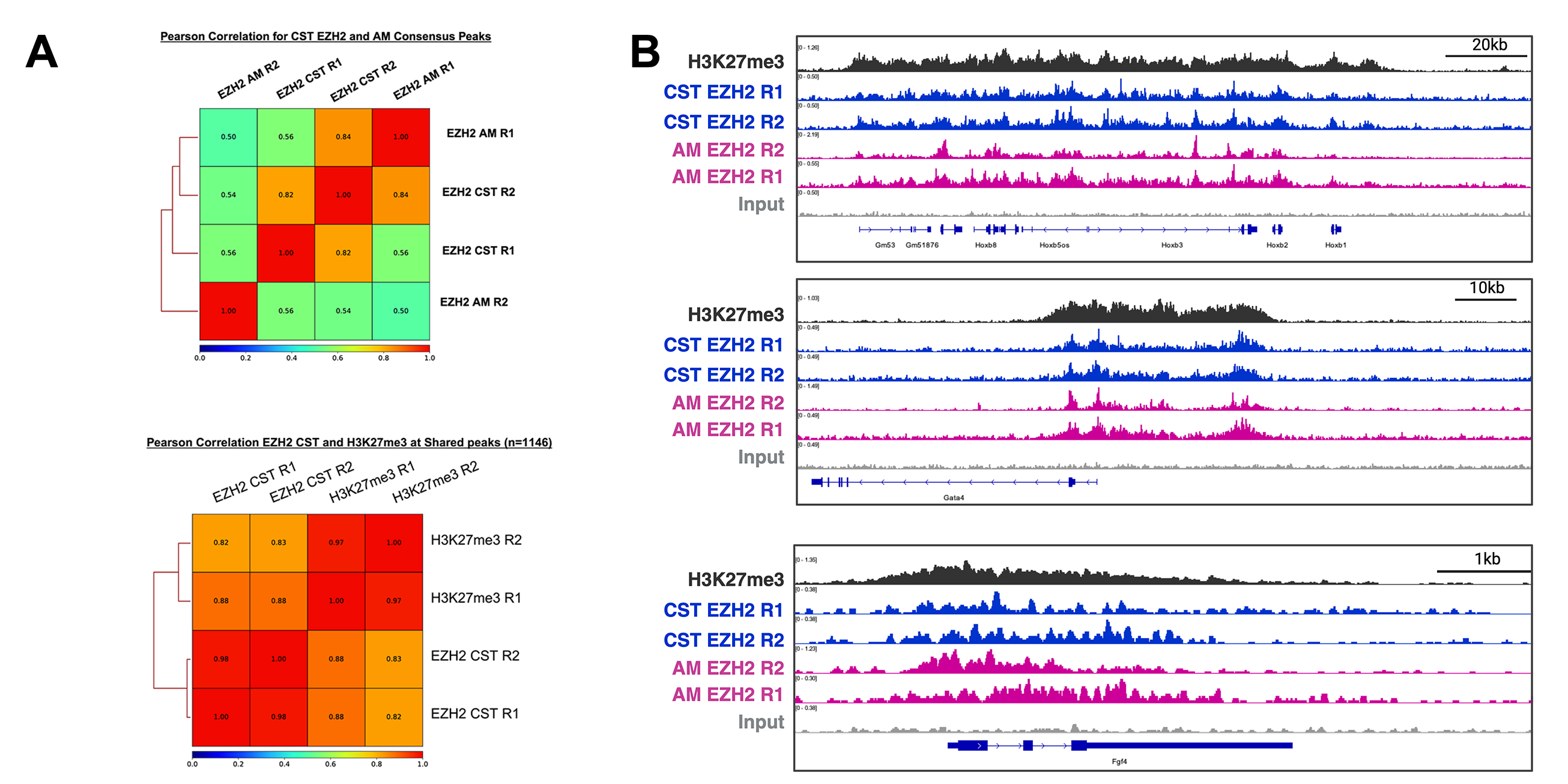
**

**Supplementary Fig. 1. ChIP binding profile for EZH2 antibodies**

(A) Pearson correlation heatmaps showing reproducibility between EZH2 ChIP (CST) and EZH2 AM datasets across biological replicates at consensus EZH2 peaks (top) and at peaks shared with H3K27me3 (bottom).

(B) Genome browser track highlighting shared binding profile for both CST and AM EZH2 samples with input control shown in gray.


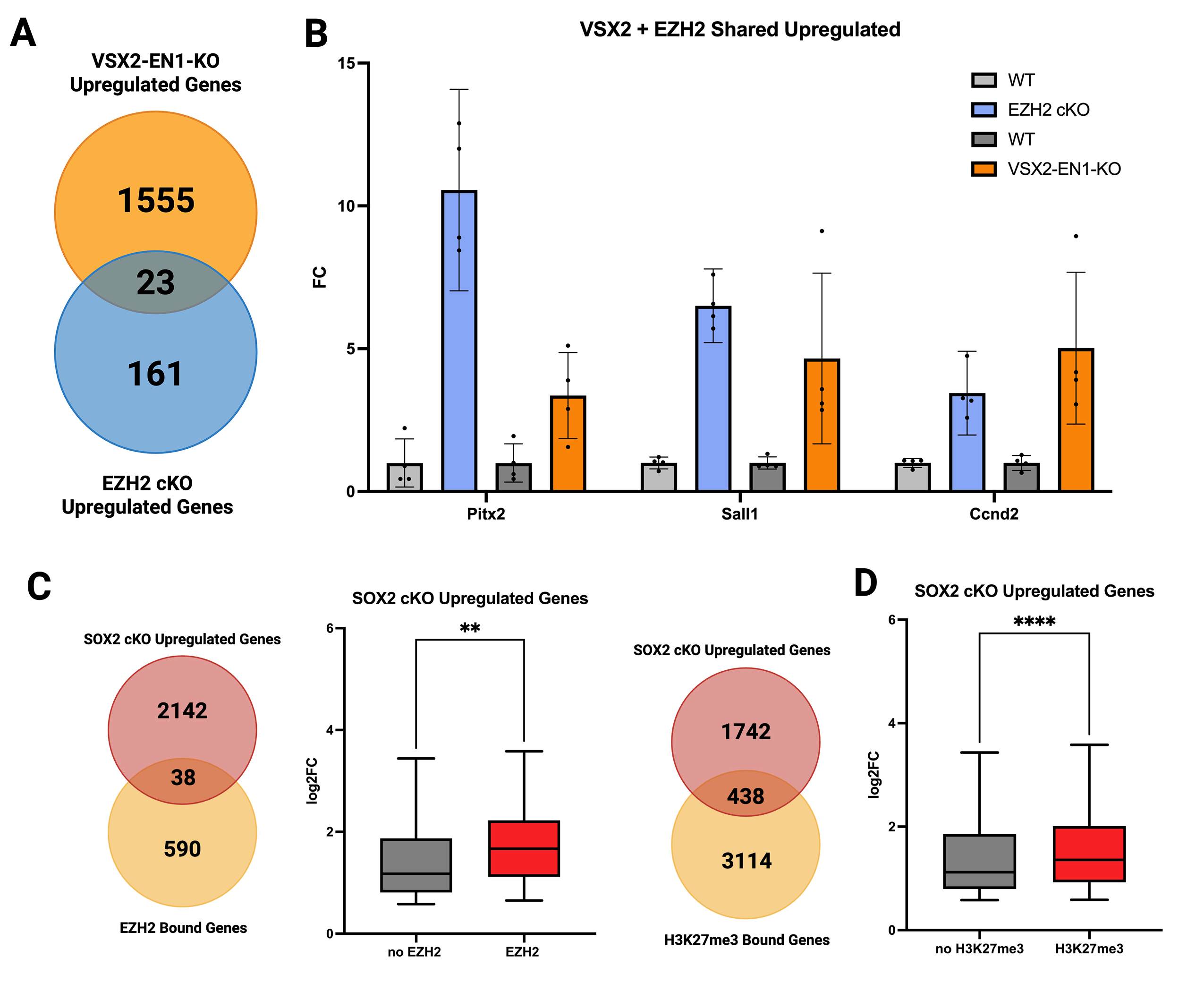


**Supplementary Fig. 2. SOX2-regulated genes show sensitivity to polycomb-mediated repression**

(A) Venn diagram illustrating the overlap between genes upregulated in VSX2-EN1-KO and EZH2 cKO retina at E14.5.

(B) Bar graph showing fold change (FC) in expression of representative genes that are commonly upregulated in VSX2-EN1-KO and EZH2 cKO samples. Data are presented as mean ± SD, with individual data points shown.

(C) Intersection of significantly upregulated genes from SOX2 cKO and EZH2 cKO (left). Box-and-whisker plot (right) shows log2 fold change (log2FC) of SOX2 cKO–upregulated genes stratified by EZH2 binding status.

(D) Venn diagram showing the overlap between SOX2 cKO–upregulated genes and H3K27me3-bound genes (left). Corresponding box-and-whisker plot (right) shows log2FC of SOX2 cKO–upregulated genes with or without H3K27me3 occupancy.
